# Expression of *RASSF1A*, *DIRAS3*, and *AKAP9* Genes in Thyroid Lesions: Implications for Differential Diagnosis and Prognosis of Thyroid Carcinomas

**DOI:** 10.3390/ijms25010562

**Published:** 2024-01-01

**Authors:** Kamila Soboska, Michał Kusiński, Karol Pawelczyk, Monika Migdalska-Sęk, Ewa Brzeziańska-Lasota, Karolina H. Czarnecka-Chrebelska

**Affiliations:** 1 Department of Biomedicine and Genetics, Medical University of Lodz, 251 Str. Pomorska, 92-213 Lodz, Polandmonika.migdalska-sek@umed.lodz.pl (M.M.-S.);; 2 Department of Oncobiology and Epigenetics, Faculty of Biology and Environmental Protection, University of Lodz, Pomorska 141/143, 90-236 Lodz, Poland; kamila.soboska@biol.uni.lodz.pl; 3 Department of Endocrine, General and Vascular Surgery, Medical University of Lodz, 62 Str. Pabianicka, 93-513 Lodz, Poland; michal.kusinski@umed.lodz.pl; 4 Faculty of Medicine, Medical University of Lodz, Av. Kościuszki 4, 90-419 Lodz, Poland

**Keywords:** thyroid carcinoma, thyroid nodules, signalling pathways, *BRAF* V600E, *RASSF1A*, *DIRAS3*, *AKAP9*

## Abstract

Thyroid carcinoma is the primary endocrine malignancy worldwide. The preoperative examination of thyroid tissue lesion is often unclear. Approximately 25% of thyroid cancers cannot be diagnosed definitively without post-surgery histopathological examination. The assessment of diagnostic and differential markers of thyroid cancers is needed to improve preoperative diagnosis and reduce unnecessary treatments. Here, we assessed the expression of *RASSF1A*, *DIRAS3*, and *AKAP9* genes, and the presence of *BRAF* V600E point mutation in benign and malignant thyroid lesions in a Polish cohort (120 patients). We have also performed a comparative analysis of gene expression using data obtained from the Gene Expression Omnibus (GEO) database (307 samples). The expression of *RASSF1A* and *DIRAS3* was decreased, whereas *AKAP9*’s was increased in pathologically changed thyroid compared with normal thyroid tissue, and significantly correlated with e.g., histopathological type of lesion papillary thyroid cancer (PTC) vs follicular thyroid cancer (FTC), patient’s age, tumour stage, or its encapsulation. The receiver operating characteristic (ROC) analysis for the more aggressive FTC subtype differential marker suggests value in estimating *RASSF1A* and *AKAP9* expression, with their area under curve (AUC), specificity, and sensitivity at 0.743 (95% CI: 0.548–0.938), 82.2%, and 66.7%; for *RASSF1A*, and 0.848 (95% CI: 0.698–0.998), 54.8%, and 100%, for *AKAP9*. Our research gives new insight into the basis of the aggressiveness and progression of thyroid cancers, and provides information on potential differential markers that may improve preoperative diagnosis.

## 1. Introduction

Thyroid carcinoma is the ninth most frequently detected cancer disease and the primary endocrine malignancy worldwide, with the most often recognised histological types (95%) derived from thyroid follicular cells: papillary thyroid cancer (PTC) and follicular thyroid cancer (FTC) [1,2,3,4]. Thyroid cancer incidence has grown in many different populations over the last three decades and is three times higher in women than men [4,5]. The development of technology and the introduction of new diagnostic methods have led to an increase in the detection frequency of non-cancerous lesions, which contribute to the phenomenon of overdiagnosis and the existence of an “alleged” thyroid cancer epidemic [2,3,4,5]. The preoperative examination of detected thyroid tissue lesions—e.g., routinely used fine-needle aspiration biopsy (FNAB)—is often unclear. In approximately 25% of FNABs, the diagnosis cannot be established before surgical excision, and only subsequent post-operational examination provides an unequivocal diagnosis [6]. Ultimately, only 10–40% of indeterminate FNABs turn out as malignant lesions in subsequent histopathological reports [7,8], leading to overestimation of the statistics regarding thyroid cancer epidemiology. At present, three genetic panels for the preoperative evaluation of thyroid nodules are available: Affirma^®^ GSC, ThyGenX/ThyraMIR^®^ ThyraGeNEXT/ThyraMIR^®^, and Thyroseq v3^®^, particularly on the USA market [9,10]. Those tests include mRNA/DNA NGS sequencing or mRNA/miRNA expression analysis. However, in Europe, these tests are only available in certain diagnostic centres and are not covered by health funds or insurance companies due to their high cost. That is why there is still an emerging need to search for accessible diagnostic and differential markers of thyroid cancers to improve preoperative diagnosis and reduce unnecessary treatments and lifelong hormone replacement therapy. Although thyroid surgery may be considered a safe procedure, it comes with significant risks, including but not limited to recurrent laryngeal nerve injury, hypocalcaemia, hematoma, scarring, and reduced quality of life [11].

**Figure 1:**
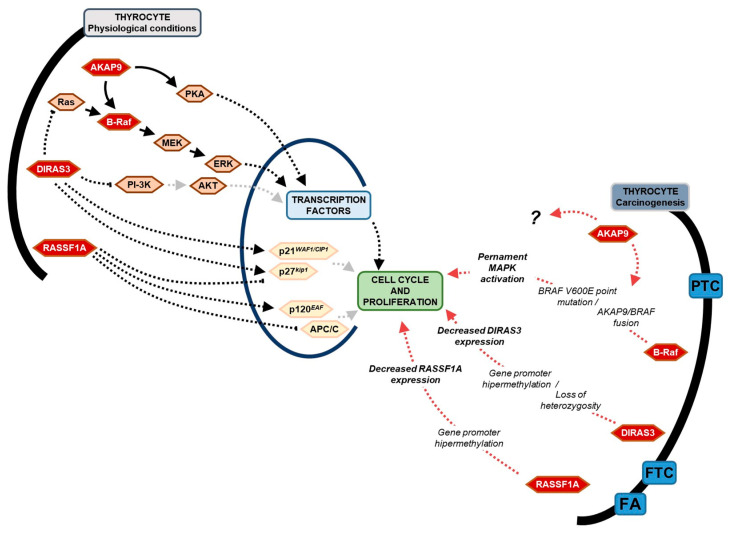
Simplified scheme of physiological action of B-raf kinase, DIRAS3, RASSF1A, and AKAP9 proteins in thyrocyte and their correlation in thyroid carcinogenesis.

Here, we focused on the potential utility of assessing the *BRAF* V600E mutation and *RASSF1A*, *DIRAS3*, and *AKAP9* genes expression in different types of thyroid tissue lesions. All aforementioned gene products are involved in signalling pathways crucial for cell cycle progression, differentiation, and cell death, and are therefore related to the pathology of tumours formation. Their simplified action in physiological conditions and in thyroid tumorigenesis is summarized and presented in Figure 1. Molecular changes in genes encoding MAPKs (Mitogen-activated protein kinases) cause constitutive activation of MAPK pathway without an external signal or affect factors acting as MAPK pathway suppressors [12,13,14,15]. Approximately 8% of all human cancers harbour the mutated *BRAF* gene, and *BRAF* V600E mutation is the most common genetic alteration in thyroid cancers within the MAPK signalling pathway. *BRAF* T1799A transversion—resulting in valine-to-glutamate substitution at codon 600 (V600E)—affects the conformation of the ATP-binding site of the protein and makes it permanently active [2,13,14,15,16,17,18,19]. Apart from genetic changes directly involved in the MAPK pathway, interesting action in thyroid carcinogenesis seem to have proteins RASSF1A and DIRAS3 (previously named ARH1—A Ras homolog member I), which are physiologically responsible for cell cycle regulation, thus giving them a tumour suppressor function [20,21]. The action of the RASSF1A protein is related to the negative regulation of Cyclin D1 activity, essential for G1 to S phase transition and accumulation of Cyclin A2 throughout the interaction with transcription factor p120^E4F^. RASSF1A also interacts with the significant cell cycle regulator Cdc20, which activates the anaphase-promoting complex (APC) [20,22,23]. The primary function of the DIRAS3 protein, belonging to the Ras protein family, is interaction with the Ras protein, impairing Ras-Raf binding, which disables constant activity of MAPK signalling [24,25]. Moreover, the DIRAS3 protein is involved in the downregulation of cyclins A and D1 activity, cyclin-dependent kinases CDK2 and CDK4, and in the enhancement of cyclin-dependent kinase CDK inhibitors like p21^WAF1/CIP1^ or p27^kip1^ [24,26,27]. DIRAS3 was pointed as a protein modulating other signalling pathways crucial for cell division related with the action of PI-3K/AKT, mTOR, NF-κB, or STAT3 [21,24,27,28,29]. Cancer-protective action of both RASSF1A and DIRAS3 are affected in thyroid cancer as a result of gene expression silencing [30], mainly hypermethylation in the promoter region and loss of heterozygosity [21,31], respectively. The next and new exciting target in cancer development and progression studies seems to be A-kinase anchoring protein 9 (AKAP9). This multifunctional protein is physiologically involved in signalling pathways crucial for cell proliferation in most human tissues [32]. The primary function of AKAP9 is interaction with components of protein kinase A (PKA) and protein phosphatase 1 and 2A signalling pathways [32,33,34]. *AKAP9* involvement in cancer disease was first mentioned in thyroid cancers, where chromatin rearrangement (paracentric inversion of chromosome 7q) results in the formation of an AKAP9-BRAF fusion protein that affects B-Raf action and constitutively activates the MAPK pathway [35]. So far, the pro-cancerous activity of AKAP9 has been extensively studied in colorectal cancer [36,37], gastric cancer [38], and acute myeloid leukaemia [39], where its action was related to cancer cell proliferation, migration, and invasion [32], as well as lower survival [37]. Since the changes in *AKAP9* expression were associated with the tumorigenesis process, we question whether they may contribute to the development of thyroid tumours. 

In our study, we initially performed the original research on tissues from the Polish cohort concerning genes selected on the basis of the data available in the literature as potentially involved in the process of thyroid carcinogenesis and useful in the diagnosis of thyroid cancer (namely *BRAF*, *RASSF1A*, *DIRAS3* and *AKAP9*). After noting statistically significant changes, possibly useful for improving the diagnosis of thyroid cancer, we decided to compare and verify them in an extended study group. Therefore, additional analyses were performed on data publicly available in the Gene Expression Omnibus (GEO) database.

## 2. Results

### 2.1. BRAF V600E Mutation

*BRAF* mutations (the presence of the mutated 144bp allele) were observed in 35.9% of all analysed samples (Supplementary materials; Result S1, Figure S1). We did not observe a significant correlation of BRAF V600E frequency with the patient’s age or gender (see Table 1, lines A and B). The percentage of V600E point mutation-positive samples in examined subtypes of thyroid lesions was highest in malignant changes: PTC (52.4%) and FTC (50.0%). The presence of the BRAF V600E mutation was also indicated in benign thyroid changes: FA (33.3%) and NG (17.1%) (Supplementary materials; Result S1, Figure S2). The frequency of V600E point mutation was significantly higher in malignant thyroid changes than in benign lesions, especially in comparison between PTC and NG (*p* = 0.004, *p* = 0.0006, respectively; Fisher exact test, see Table 1, lines C and D). The analysis of samples from PTC and FTC also indicated an insignificant increase of *BRAF* point mutation frequency along with the level of cancer progression (in larger tumours with nodules infiltration; Table 1, lines E and G). Nonetheless, the presence of *BRAF* V600E point mutation was not correlated with other pathological features of malignant samples such as the pT scale and AJCC stage (Table 1, lines F and H).

### 2.2. RASSF1A Expression

**Figure 2:**
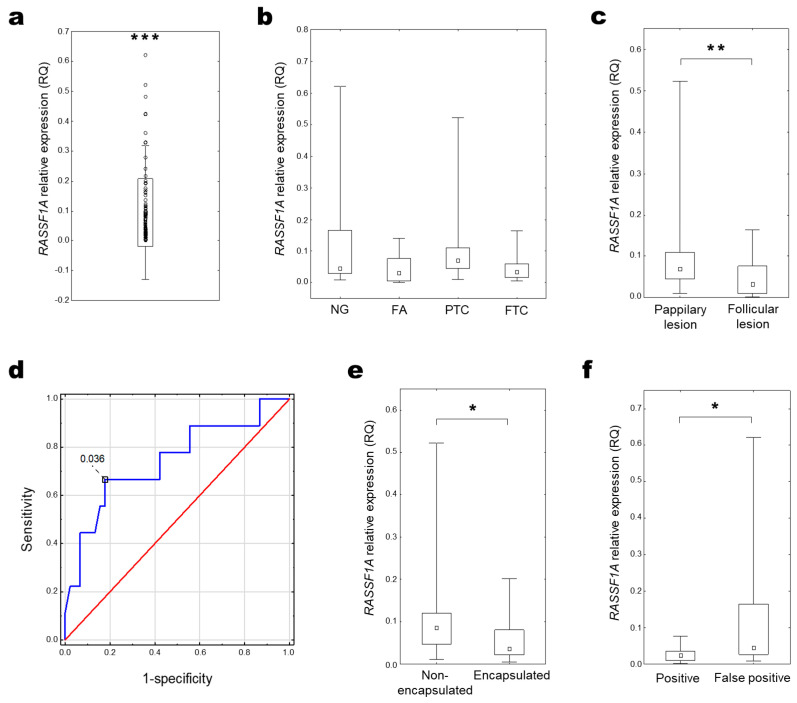
*RASSF1A* expression in thyroid tissue. (**a**) Distribution of relative *RASSF1A* expression in all examined samples (RQ) (One sample Wilcoxon test; *** *p* < 0.001). (**b**) *RASSF1A* expression compared among histopathological groups (KW test, *p* > 0.05) and (**c**) papillary- and follicular-types of thyroid lesions (UMW test; ** *p* = 0.01–0.001); (**d**) ROC curve analysis for *RASSF1A* expression in differentiation FTC from PTC, where blue line stands for actual ROC curve, and red for diagonal. *RASSF1A* expression in (**e**) non-encapsulated and encapsulated tumour groups (UMW test; * *p* = 0.05–0.01). (**f**) *RASSF1A* expression in patients regarding positive and negative (false positive) cytological verification of FNABs (UMW test; * *p* < 0.05).

The relative expression level (RQ) analysis indicated decreased expression of the *RASSF1A* gene in all pathologically changed tissues compared to control healthy thyroid tissue (median RQ = 0.055 (0.029–0.107); *p* < 0.001, One sample Wilcoxon test; Figure 2a). There was significantly decreased expression of the *RASSF1A* gene in follicular-type thyroid lesions (FA and FTC) compared to papillary thyroid lesions (PTC; Figure 2c). Moreover, analysis of the receiver operating characteristic (ROC) curve of *RASSF1A* expression revealed its utility in malignant thyroid cancer classification. The best cut-off point for FTC differentiation from PTC was equal to ≤0.036 for *RASSF1A* (*p* = 0.014; Youden’s J statistic). The area under the curve (AUC) was equal to AUC 0.743 (95% CI: 0.548–0.938), and its predictive value was as follows: specificity—82.2%, positive predictive value (PPV)—42.9%, sensitivity—66.7%, and negative predictive value (NPV)—92.5% (Figure 2d).

Lower *RASSF1A* expression was also observed in encapsulated tumours compared with non-encapsulated ones (Figure 2e). In patients with initial FNAB diagnosis of follicular neoplasm (Bethesda category IV), the *RASSF1A* expression was significantly decreased in samples with further positive histopathological verification (confirmed FA and FTC) compared to false positive results (particularly NG; Figure 2f).

Additionally, in the conducted multivariate analysis of the co-presence of mutations and genetic instabilities, we have demonstrated that the presence of LOH/MSI in the *RASSF1A* region and *BRAF* V600E mutation both impact *RASSF1A* expression. Moreover, in patients with a malignant change, the presence of the *BRAF* mutation resulted in higher *RASSF1A* expression than those without *BRAF* mutations (*p* = 0.048; Kruskal–Wallis rank sum test, see Supplementary materials; Result S3).

### 2.3. DIRAS3 Expression

**Figure 3:**
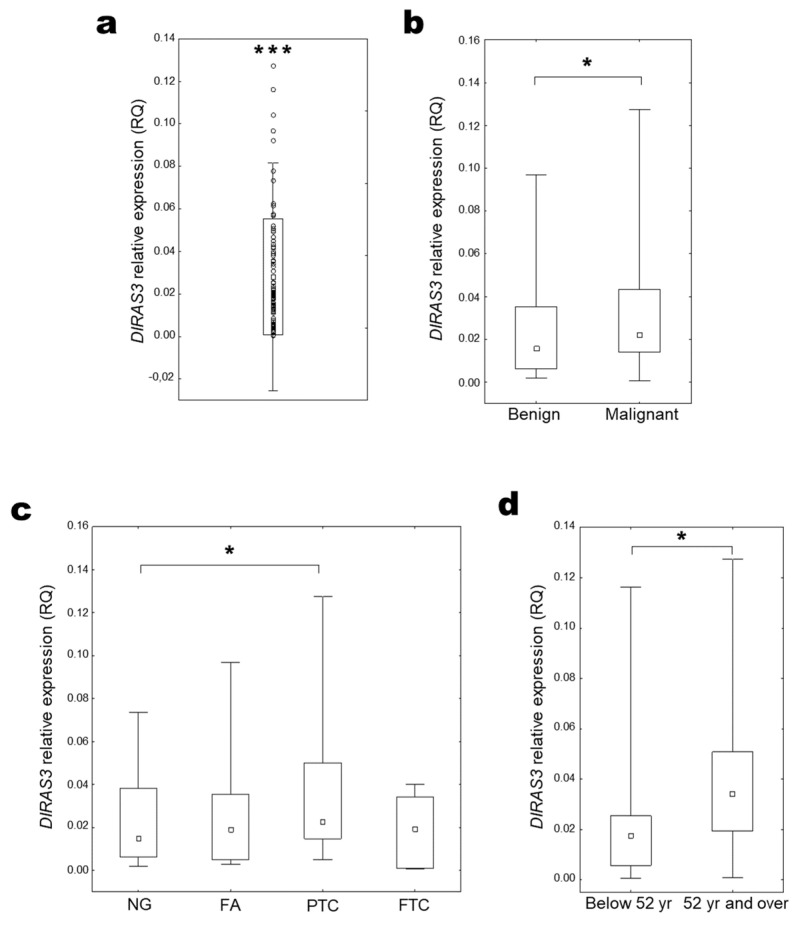
*DIRAS3* expression in thyroid tissue. (**a**) Distribution of relative *DIRAS3* expression in all examined samples (RQ) (One sample Wilcoxon test; *** *p* < 0.001). (**b**) *DIRAS3* expression compared among benign and malignant lesions (UMW test, * *p* = 0.05–0.01) and (**c**) among histopathological groups (KW test, * *p* = 0.05–0.01); (**d**) *DIRAS3* expression in patients with malignant lesions depending on the median age (UMW test, * *p* = 0.05–0.01).

Analysis of obtained results indicated a decreased expression of the *DIRAS3* gene in all pathologically changed tissue compared to the control healthy thyroid tissue (median RQ = 0.019 (0.007–0.042); *p* < 0.001, One sample Wilcoxon test, Figure 3a). Comparing various clinical and pathological features of samples, the level of *DIRAS3* gene expression was significantly higher in malignant thyroid changes (FTC and PTC) than in benign lesions (NG and FA) (Figure 3b). The highest expression level was observed in PTC samples, and at a comparable level in other examined histopathological types (Figure 3c). Increased *DIRAS3* expression was also observed in the group of older patients with malignant lesions (Figure 3d).

### 2.4. AKAP9 Expression

**Figure 4:**
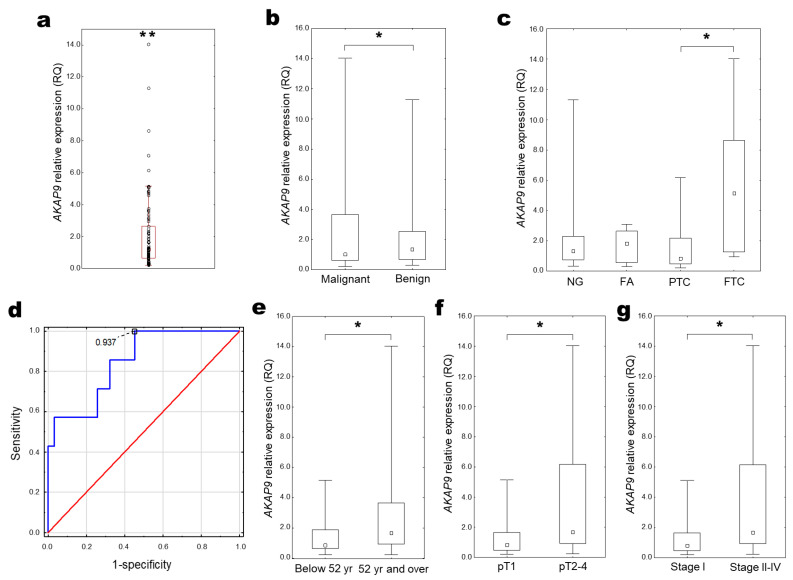
*AKAP9* expression in thyroid tissue. (**a**) Distribution of relative *AKAP9* expression in all examined samples (RQ) (One sample Wilcoxon test; ** *p* = 0.01–0.001). (**b**) *AKAP9* expression compared between benign and malignant lesions (UMW test, * *p* = 0.05–0.01) and (**c**) histopathological groups (KW test, * *p* = 0.05–0.01). (**d**) ROC curve analysis for *AKAP9* expression in differentiation FTC from PTC, where blue line stands for actual ROC curve, and red for diagonal. *AKAP9* expression depending on the: (**e**) median age; (**f**) tumour size; and (**g**) tumour stage (UMW test, * *p* = 0.05–0.01).

Analysis of obtained results indicated an increased expression of the *AKAP9* gene in all pathologically changed tissue compared to the control healthy thyroid tissue (median RQ = 1.209 (0.646–2.727); *p* = 0.01–0.001, One sample Wilcoxon test, Figure 4a). Although expression level was generally higher in benign thyroid changes in comparison with malignant lesions (Figure 4b), the highest expression was indicated in FTC samples (Figure 4c). The *AKAP9* expression was significantly increased in FTC samples compared with PTC samples (Figure 4c). The utility of the *AKAP9* expression estimation for classifying malignant thyroid cancer was confirmed with the ROC curve analysis. The best cut-off point for FTC detection was equal to ≤0.94 for *AKAP9* (*p* < 0.001; Youden’s J statistic). The area under the curve (AUC) was equal to AUC 0.848 (95% CI: 0.698–0.998), and its predictive value was as follows: specificity—54.8%, positive predictive value (PPV)—33.3%, sensitivity—100%, and negative predictive value (NPV)—100% (Figure 4d).

Increased *AKAP9* expression was revealed to correlate with older age (Figure 4e) and more advanced tumours (pT2-4 vs. pT1 and stage II-IV vs. I; Figure 4f,g).

### 2.5. RASSF1A, DIRAS3, and AKAP9 Expression in Thyroid Tissue (in GEO Database)

Expression of *RASSF1A*, *DIRAS3,* and *AKAP9* genes was also analysed using data obtained from the GEO database, including 227 pathologically changed tissues (35 FA, 44 FTC and 148 PTC) and 80 control non-cancerous tissues (including 44 samples from PTC and non-tumour control obtained from the same patients). Due to the limited data available, the analysis of the expression of all tested genes concerning the histopathological features of tissues was carried out only on a group of samples obtained from PTC.

**Figure 5:**
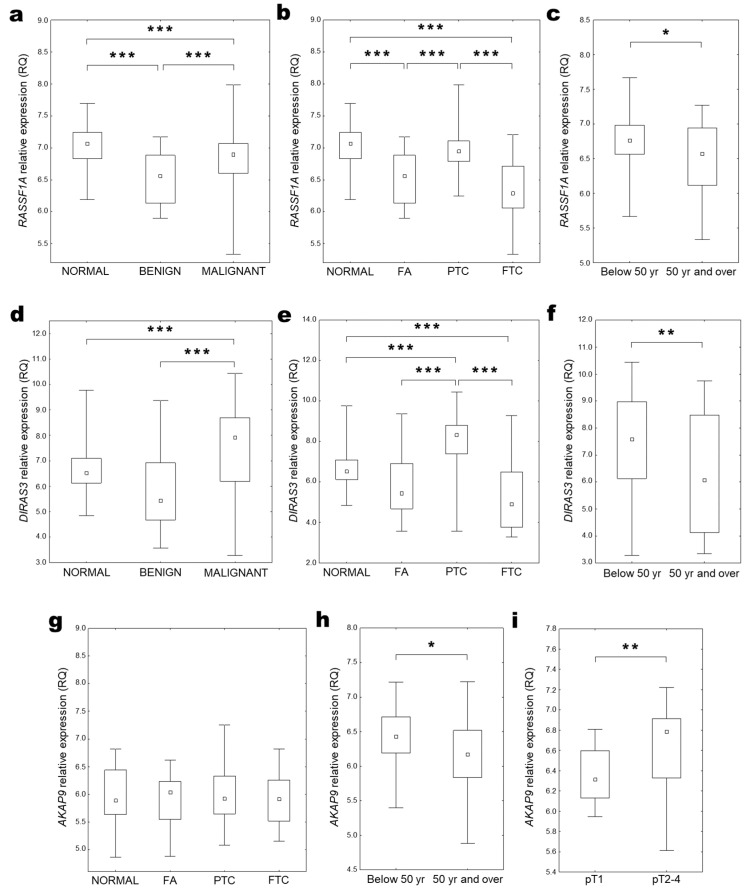
*RASSF1A* (upper row), *DIRAS3* (middle row), and *AKAP9* (lower row) expression in thyroid tissue (GEO data analysis). Gene expression compared among (**a**,**d**) benign (NG and FA) and malignant (PTC and FTC) thyroid changes, (**b**,**e**,**g**) different histopathological types of thyroid tissue lesions (KW test, *** *p* < 0.001) with unchanged thyroid tissue as the control (NORMAL); Gene expression compared among (**c**,**f**,**h**) groups of patients below (Below 50 y) and over (50 y and over) median age (UMW test, * *p* = 0.05–0.01; ** *p* = 0.01–0.001) and (**i**) cancer stages expressed with the pT scale (UMW test, ** *p* = 0.01–0.001).

**Figure 6:**
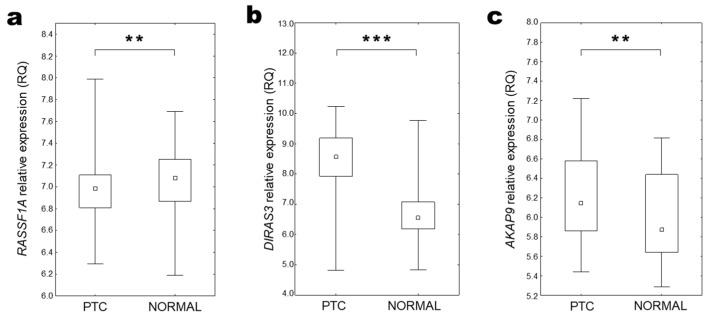
Patient-matched data analysis of gene expression in thyroid tissue (GEO data analysis). (**a**) *RASSF1A*, (**b**) *DIRAS3,* and (**c**) *AKAP9* expression in thyroid cancer (PTC) compared with patient-matched unchanged thyroid tissue samples (NORMAL) (Wilcoxon matched-pairs signed rank test; ** *p* = 0.01–0.001; *** *p* < 0.001).

Analyses performed using all available tissues indicated that *RASSF1A* expression was decreased in both benign and malignant tissue lesions compared to control samples (Figure 5a). Comparing various histopathological types of thyroid tissue changes, the lowest *RASSF1A* expression was observed in FTC and FA samples (Figure 5b). Although in the cumulative analysis the median *RASSF1A* expression level in PTC was comparable with expression in normal tissue (Figure 5b), the patient-matched analysis proved a significantly lower expression level also in this type of thyroid cancer (Figure 6a). A lower *RASSF1A* expression was also observed in older patients (over median age equal 50 years; Figure 5c).

*DIRAS3* expression was decreased in benign tissue changes and increased in malignant thyroid lesions in comparison to normal thyroid tissue (Figure 5d). However, a more detailed analysis of the available data showed decreased expression in FA and FTC samples, and increased expression only in PTC samples (Figure 5e and Figure 6b). Lower *DIRAS3* expression was also observed in older patients (over the median age of 50 years; Figure 5f).

Cumulative analysis of *AKAP9* gene expression did not show significant changes in comparison between benign and malignant tissue lesions with control tissue or in comparison between different types of thyroid changes (Figure 5g). However, patient-matched data analysis revealed higher expression levels in PTC samples compared to normal thyroid tissue (Figure 6c). *AKAP9* expression was also increased in older age patients (over median age equal 50 years; Figure 5h) and in samples obtained from the more advanced tumour (characterised by pT3 and pT4 parameters; Figure 5i).

## 3. Discussion

According to the latest edition of the Global Cancer Observatory GLOBOCAN 2020, there are 586,000 cases of thyroid cancer and 44,000 deaths each year [5]. Although thyroid cancer has been extensively studied and has a relatively low mortality rate, diagnosing this type of cancer remains challenging, leading to overtreatment and frequent unnecessary thyroidectomy [2,3,4]. Identifying factors that can facilitate early cancer diagnosis, differentiate between types of lesions, or be low-cost markers of a more severe disease course remains a fundamental research issue. Our studies on tissues from patients with benign and malignant thyroid lesions showed a significant contribution of the *BRAF* V600E mutation and changes in the expression of *RASSF1A*, *DIRAS3*, and *AKAP9* genes in developing this type of cancer. Therefore, we conducted further analyses on the extended dataset from five experiments available in the GEO databases to confirm the obtained results. Our research approach is based on the examination of various types of changes present in the thyroid glands, not only the most frequently studied cancers as PTC and FTC, but also not cancerous NG nor benign FA. Hence, it was possible to compare molecular events occurring in benign and malignant thyroid lesions and assess their potential as differentiation markers, which could be useful in evaluating the need for a thyroidectomy.

One of the most described genetic alterations leading to thyroid carcinogenesis is the *BRAF* V600E point mutation. The percentage of reported thyroid carcinoma cases with a mutated BRAF gene mainly depends on the studied population. The incidence of *BRAF* V600E varies from 19% to 83% of pathologically changed thyroid tissues [1,15,16,40,41]. Simultaneously, the method used for detecting *BRAF* V600E requires careful consideration due to the significant variation in the number of samples with *BRAF* mutations that are detected depending on the approach used. For instance, Brzezianska et al. discovered a higher frequency of *BRAF* V600E mutations when using single-strand conformation polymorphism and a real-time allele-specific PCR (AS-PCR) assay compared to direct sequencing [17]. Kim et al. compared the RT-PCR and pyrosequencing sensitivity in *BRAF* V600E mutation detection in thyroid FNABs. The RT-PCR showed higher sensitivity (61.9% vs. 57.8%) but lower specificity (78.6% vs 85.7%) than pyrosequencing. Considering the ease and speed of testing using RT-PCR methods, it was postulated as more convenient than pyrosequencing (indicating the need for careful interpretation criteria to balance sensitivity and specificity in detecting the *BRAF* V600E mutation) [42]. In the present study, we used amplification refractory mutation system PCR (ARMS-PCR) analysis that allowed us to find the *BRAF* V600E mutation in approximately 60% more samples than using the Sanger sequencing (Supplementary materials; Method S1 and Result S1, Figures S1 and S2). Similarly, Ellison et al. found that the ARMS-PCR technique was more sensitive and robust than Sanger sequencing in detecting somatic mutations in clinical samples [43]. As demonstrated in a study by Huang et al. (2013), the ARMS-PCR is a highly sensitive method enabling the detection of the mutated *BRAF* V600E allele in 0.5% wild background [15]. This may explain the higher sensitivity of *BRAF* V600E mutation detection with ARMS-PCR compared to Sanger sequencing. Interestingly, one of the mutations detected with ARMS-PCR was subsequently confirmed as a K601E point mutation in Sanger sequencing (Supplementary materials; Result S1, Figure S2b). The detection of both V600E and V600K using the ARMS-PCR assay for *BRAF* 1799T>A mutation has been previously described in lung cancer [43].

Nevertheless, even considering the high discrepancy in percentage of *BRAF* V600E mutated samples, this point mutation is considered a hallmark of thyroid cancer, especially characteristic for its papillary variant [44]. Importantly, most of the published works analyse tissue samples excised from a single type of cancer, primarily focusing on the most common PTC and its subtypes. At the same time, studies comparing different histopathological subtypes of thyroid cancers and benign lesions are uncommon. Our study identified *BRAF* V600E mutation in 35.9% of all examined samples. However, we assessed the frequency of mutations not only in neoplastic lesions such as PTC and FTC, but also in benign lesions, including FA and NG. Contrary to the majority of previously published work [1,15,44], we showed a comparable rate of *BRAF* V600E frequency both in PTC (52.4%) and FTC (50.0%) derived samples. Unusually, the *BRAF* V600E mutation occurrence was also observed not only in the malignant lesions, but also in benign FAs (33.3% of samples) and noncancerous but pathologically changed NGs (17.1%). That may indicate its character of an early event present in the precancerous stage. Simultaneously, there was a significantly higher percentage of *BRAF*-mutated samples in malignant changes compared with benign lesions, which suggests its potential as a differential marker, referring the patient for surgery. More detailed analyses have shown that assessing the presence of this marker will be the most effective in differentiating between NG and PTC. Hypothetically, the presence of the mutation in NG may indicate precancerous changes without detectable changes in cell phenotype, and it could be utilized in oncological risk evaluation [45]. The results of studies on the frequency of V600E mutations, depending on the clinical and pathological characteristics of the patients, still remain controversial. While some authors show no correlation between the increased frequency of *BRAF* V600E mutations for one or more of the above parameters, others indicate a higher occurrence of mutation in older patients [1,44,46,47] and in tissues from cancers characterised by more advanced stage [1,18,46,47], larger size [1,46], or presence of lymph nodes metastases [1,18,47,48]. Based on our study, there is a tendency for an increase in the percentage of *BRAF* mutations in tumour samples obtained from older patients or those with larger tumours or nodular infiltration.

Expression of the *RASSF1A* tumour suppressor gene is known to be downregulated as an effect of various epigenetic mechanisms. Detection of such molecular events is frequent across multiple types of cancer, including liver cancer (90% of cases), small cell lung cancer (80%), prostate cancer (70%), and non-small cell lung cancers (52%) [20,49,50]. In thyroid tumours, downregulation of *RASSF1A* expression has also been reported so far and is mainly correlated with inappropriate promoter methylation [30,49,51,52,53]. In our studies, alterations of *RASSF1A* expression and hypermethylation of its promoter region were observed in all histopathological groups of thyroid lesions (Supplementary materials; Result S2). According to numerous published studies on thyroid cancers, the *RASSF1A* methylation and therefore silencing of gene expression correlated with more aggressive cancer phenotype/subtype—i.e., follicular, medullary, and undifferentiated forms (70–80%)—but was less often in PTC (15–63%) [30,51,52,53]. Hence, some studies suggest that assessing *RASSF1A* hypermethylation can help distinguish FTC from FA [54]. Interestingly, *RASSF1A* gene methylation has been detected in non-cancerous tissue near the primary PTC lesion [52,55], indicating possible molecular-level changes related to early cancer development. Moreover, the silencing of *RASSF1A* via promoter hypermethylation occurred with lower frequency in the early stages of carcinogenesis (FA) [52,53], as well as in non-cancerous proliferative changes like nodular goitres. Our results partially align with previous research, as the expression of *RASSF1A* was observed to be more extensively decreased in follicular-type lesions (FA and FTC) compared to the papillary-type of lesions. Strongly decreased expressions of *RASSF1A* can be treated as a hallmark of follicular thyroid cancer, with 82.2% specificity and 66.7% sensitivity (AUC 0.743). The *RASSF1A* RQ value over 0.036 may indicate PTC; hence, assessing the expression can help distinguish PTC from FTC. Simultaneously, our study also investigated gene expression regarding the initial FNAB diagnosis. In patients with follicular neoplasm (Bethesda category IV) confirmed in post-surgery histopathological verification, the *RASSF1A* expression was significantly decreased compared to patients with false positive results (mainly the presence of NG). This result seems promising, as it could support differential diagnosis before surgery, seeing that misdiagnosis of nodular goitres (in case of indeterminate FNABs) leads to unnecessary thyroidectomy in a particular proportion of patients. The epigenetic *RASSF1A* silencing was also detected in follicular thyroid hyperplasia (FTH)—cellular hyperplasia leading to the enlargement of the thyroid gland. The *RASSF1A* downregulation correlated with NF-κB activation present in a subset of FTHs may indicate a potential for progression to malignancy [56]. Numerous studies suggested a relationship between the degree of promoter hypermethylation *RASSF1A* and tumour grade, distant metastases [51], and extracapsular invasion [55]. Our research has revealed that *RASSF1A* silencing occurs more frequently in samples with lymph node infiltration (N1) than in those without nodule involvement (N0). Strikingly, we noticed significantly lower *RASSF1A* expression in encapsulated tumours. Encapsulated PTC is known to have a better prognosis (lower risk of recurrence and improved overall prognosis) than non-encapsulated PTC. Thus, *RASFF1A* silencing in the case of encapsulated lesions could be regarded as a favourable prognostic marker for thyroid tumorigenesis. The silencing of *RASSF1A* through hypermethylation was postulated to be exclusive to *BRAF* V600E mutations [53,57,58]. In our study, we discovered that *RASSF1A* expression is impacted by the co-occurrence of LOH/MSI in the *RASSF1A* region and the *BRAF* V600E mutation (Supplementary materials; Result S3). Similar correlations of decreased *RASSF1A* expression and LOH/MSI presence have already been suggested in other cancers as neuroblastoma [59] or prostate cancers [60].

*DIRAS3* is a tumour suppressor gene that plays a role in cell proliferation, apoptosis, and tumour development. Expression of *DIRAS3* has been found to be dysregulated in various types of cancers, including thyroid neoplasms. A decrease of the DIRAS3 expression was found particularly in FTC, but not in PTC [30,31]. Analysis of data available in GEO database confirmed the changes published in the mentioned literature, indicating the differentiating potential of the examined factor. Simultaneously, downregulation of *DIRAS3* expression has not been previously observed in benign thyroid lesions, such as nodular goitres (NG) or follicular adenoma (FA) [30,31], and our detailed comparisons of *DIRAS3* expression level revealed a significant decrease not only in benign lesions (compared to malignant ones), but also in NG compared to PTC. *DIRAS3* expression in FA and FTC was on a comparable level. Since the *DIRAS3* gene is monoallelic maternally imprinted, we did not assess the presence of the *DIRAS3* promoter hypermethylation in this study. Additional studies performed in selected GEO datasets confirmed *DIRAS3* expression decrease in benign lesions, while an increase was observed in malignant thyroid lesions (mainly PTC) in comparison to normal thyroid tissue. Both analyses showed a *DIRAS3* expression decrease in FA and FTC when compared to PTC, indicating the putative usefulness of this gene in the differentiation analysis. In Zhu and Qu’s study, the immune-expression levels of DIRAS3 (ARHI) and Beclin1 proteins in thyroid cancer tissues were significantly lower than in adjacent tissues. Patients with DIRAS3 and Beclin1 low-expression had significantly lower three-year survival rates than patients with high expression levels [61]. This suggests that the expression levels of these genes may have prognostic value in thyroid cancers. Some studies have shown that the decrease in *DIRAS3* expression is more prominent in younger patients with thyroid cancer, indicating the potential role of *DIRAS3* in the aggressive behaviour of thyroid tumours in certain age groups [21,31]. Concordantly with this finding, in our studies on the Polish cohort, *DIRAS3* expression was significantly lower in a group of patients below median age (however, analysis of data from the GEO database gave the opposite results). Another interesting observation regarding the *DIRAS3* gene in carcinogenesis comes from breast cancer studies, where downregulation of its expression correlated with brain metastases. In differential gene analysis performed on TCGA data containing 335 differentially expressed genes in breast cancer (with and without distant metastasis), *DIRAS3* was included in the 5-gene LASSO prognostic model [62]. The exact mechanisms behind the dysregulation of *DIRAS3* in different cancers, including thyroid, are not fully understood. Still, it is believed to involve various genetic and epigenetic alterations that affect its expression.

Various genetic alterations of the *AKAP9* are known to be primarily associated with cancers of the colorectum [34,39], stomach [34,38], lungs [63], and breast [64]. Approximately 10 to 18% of samples carrying the mutated *AKAP9* gene are reported, depending on the type of mutation and type of cancer. The studies published thus far also show upregulation of *AKAP9* gene expression in gastric [38] and CRC [36,37] cancers, as well as in acute myeloid leukaemia [39]. So far, the only known association of the *AKAP9* gene with thyroid cancer was the effect of chromatin rearrangement and the formation of an *AKAP9-BRAF* fusion protein with elevated kinase activity [35]. Here, we have analysed for the first time whether the *AKAP9* gene expression is related to the development or progression of thyroid cancer, as observed in CRC, gastric cancer, or leukaemia. Our analyses based on benign and malignant thyroid lesion tissues have revealed a significant difference in the expression of the *AKAP9* gene between healthy and cancerous thyroid tissue. Particularly, differences were noticed in paired comparison of expression data available in the GEO database concerning PTC and normal thyroid tissue. Moreover, ROC curve analysis demonstrated potential of *AKAP9* analysis in distinguishing FTC from PTC with 54.48 specificity and 100% sensitivity (AUC 0.848). *AKAP9* expression increase over 0.94 may indicate FTC, so it can be treated as a hallmark of a more aggressive thyroid cancer. Previous research has shown that increased expression of the *AKAP9* gene is associated with the proliferation, migration, and invasion of cancer cells, and therefore cancer progression and tumour metastasis [37]. Moreover, in paediatric AML, *AKAP9* overexpression was considered a marker of poor prognosis [39]. In thyroid carcinoma, increased expression of *AKAP9* is correlated with more advanced tumours (expressed by the pT scale and the AJCC scale) and older age of patients. Such observations resulted from both the analysis of tissues we collected, and the data previously shared by other research teams in the GEO database. The way AKAP9 acts in cancers still needs to be better understood. In vitro studies of molecular events associated with *AKAP9* gene expression alteration in tumour cells linked it to the action of proteins such as CDH1 [38], Cdc42 interacting protein 4 [37], and the Wnt/β-Catenin signalling pathway [39]. In thyroid cancers, the mechanism of increased expression of *AKAP9* and its influence on tumour development has not yet been elucidated. Based on our observation of increased expression of the *AKAP9* gene in more advanced tumours, it can be assumed that this is not an early event of the carcinogenesis process. At the same time, as in other types of cancer, later *AKAP9* expression alterations promote tumour development and invasiveness of tumour cells.

The search for markers enabling preoperative differentiation of thyroid cancers from benign hyperplastic lesions, as well as facilitating the differentiation of various cancer types/subtypes, is still an important research issue. The example of the *AKAP9* gene, which was previously not associated with the process of thyroid carcinogenesis—but is indicated in our work for the first time as potentially useful in the diagnosis of this cancer—shows that the search for genes with differential expressions in thyroid cancer and benign non-cancerous lesions (not requiring invasive treatment methods) is still needed. Simultaneously, the development of lesion-specific differentiation markers useful in preoperative diagnosis requires the collection of a significant number of samples from classified, pathologically changed tissues (malignant as same as benign lesions) due to the lack of such data in publicly available databases.

## 4. Materials and Methods

The procedures used in the study were approved by the Bioethical Committee of the Medical University of Lodz, Poland (Resolution no. RNN/217/11/KE). The study was conducted following the Declaration of Helsinki. All participants signed an individual consent form, in the case of 1 minor patient—consent of his legal guardian. The study was funded by the Ministry of Science and Higher Education “Iuventus Plus” (grant no. 0082/IP1/2011/71).

### 4.1. Thyroid Tissues

Thyroid tissue material was obtained from 120 patients who had undergone a total thyroidectomy performed in the Department of Endocrine, General, and Vascular Surgery, Chair of Endocrinology, Medical University of Lodz, Poland, during 2012–2020. This was a prospective study conducted at a single centre. The median age in the patient group was 52 years (range of age: 16–76). Patients were referred for surgery based on the initial FNAB diagnosis (performed in endocrine clinics appropriate to the patient’s place of residence) and according to “Diagnosis and treatment of thyroid cancer—Polish guidelines” [65]. For this study, we have included patients with the following Bethesda System for Reporting Thyroid Cytopathology (BSRTC) results: follicular neoplasm (Bethesda IV), suspicious for malignancy (Bethesda V), and malignant (Bethesda VI). We have also included non-malignant lesions (Bethesda II) patients qualified for surgery due to additional indications such as giant nodular goitre (NG), retrosternal goitre, airway compression, or thyrotoxic hyperactivity of the NG. (Results of cytological verification of FNABs are summarized in Supplementary materials; Table S1). The resected tumours were classified according to the American Joint Committee on Cancer (AJCC) 7th edition pTNM (pathological tumour-node-metastasis classification) classification system [66]. The demographic characteristics of the patients, clinical information, and histopathological information (type of lesion, pTNM classification, AJCC staging, multifocality) were obtained from pathomorphological reports and are presented in Table 2 and Table 3. During surgery, none of the patients enrolled were found to have any metastases (M0 in pTNM classification).

The thyroid tissue samples (100–150 mg) were obtained following the pattern: one sample from the centre of the primary lesion and another sample of matching noncancerous tissue (macroscopically unchanged) from the other lobe—used as a control in the study. Immediately after resection, total tissue samples were collected in a stabilization buffer RNAlater^®^ (Qiagen, Hilden, Germany) and frozen at −80 °C until further use.

### 4.2. DNA and RNA Isolation, Reverse Transcription

Extraction of genomic DNA was performed using QIAamp DNA Mini Kit (Qiagen, Hilden, Germany) according to the manufacturer’s protocol. The RNA residue was removed using the RNAse A solution (Qiagen, Hilden, Germany). The extracted DNA concentration and quality were assessed spectrophotometrically using BioPhotometer™ Plus (Eppendorf, Hamburg, Germany). DNA samples with a 260/280 nm ratio in the range of 1.8–2.0 were selected for further analysis. Total RNA was isolated using the Universal RNA Purification Kit (Eurx, Gdansk, Poland) according to the manufacturer’s protocol. RNA Integrity (RIN) was routinely evaluated using a 2100 Bioanalyzer and RNA 6000 Pico/Nano LabChip kit (Agilent Technologies, Santa Clara, CA, USA). RIN score > 7 was considered sufficient for subsequent analysis. In order to perform *BRAF* V600E mutation and gene expression analysis, 1 µg of RNA was reverse transcribed into cDNA with a High-Capacity cDNA Reverse Transcription kit (Applied Biosystems, Waltham, MA, USA).

### 4.3. ARMS-PCR

The presence of V600E substitution in exon 15 of the *BRAF* gene was evaluated in 103 pairs of thyroid specimens with Amplification Refractory Mutation System PCR (ARMS-PCR) according to Huang et al., 2013 [15]. Four different primers were used (Sequences of primers and the expected length of allele-specific products are summarised in Supplementary materials; Table S2). Two of them were designed to amplify a fragment of 200 bp flanking the mutation site, i.e., universal forward (Fo) and universal reverse (Ro); and two internal primers amplifying potential mutation site, i.e., specifically binding to the wild-type (Fiwt) or mutated (Rimut) *BRAF* gene sequence.

ARMS-PCR was performed using the Hot Start AmpliTaq 360^®^ Polymerase (5 U/µL), 5.0 µM µM of Fo, 2.5 µM of Ro and Fiwt, and 10.0 µM of Rimut primers (Sigma-Aldrich, St. Louis, MO, USA). The amplification products were separated on 2% agarose gel, stained with ethidium bromide (0.5 mg/mL), and visualized under UV light on a DigiDoc-It Imaging System (Ultra-Violet Products Ltd., Upland, CA, USA). The samples with a 144 bp product were recognized as positive for the *BRAF* V600E mutation, as well as cases where two allele-specific products were detected (144 bp for mutation and 97 bp for wild-type) [15]. Samples with the presence of products indicating the *BRAF* V600E mutation were submitted to further analysis in automated electrophoresis, using DNA1000 LabChip Kit, on Agilent 2100 Bioanalyzer (Agilent Technologies, CA, USA) to estimate the exact length of the products and exclude the possibility of a false positive result.

### 4.4. Evaluation of Gene Expression

*RASSF1A* (Gene ID: 11186, located in 3p21.3), *DIRAS3* (Gene ID: 9077, located in 1p31.3), and *AKAP9* (Gene ID: 10142, located in 7q21.2) expressions were evaluated by semiquantitative real-time PCR. *ACTB* (β-actin, Gene ID: 60) or *GAPDH* (glyceraldehyde-3-phosphate dehydrogenase, Gene ID: 2597) were used as endogenous controls. RNA isolated from the healthy thyroid lobe served as a reference sample in relative quantification analysis. The chromosomal location of the analysed genes and nucleotide sequences of primers used in the study are listed in Supplementary materials; Table S2.

The relative expression of *RASSF1A* and *DIRAS3* genes was analysed in 108 and 83 pairs of thyroid specimens, respectively, using Micro Fluidic Cards—TLDA (TaqMan Low-Density Arrays, Applied Biosystems, Foster City, CA, USA) in Applied Biosystems 7900HT Fast Real-Time PCR System (Applied Biosystems, CA, USA). The qPCR mix contained cDNA and TaqMan Universal Master Mix (Applied Biosystems, CA, USA). The selected TaqMan probes Hs00200394-m1 for *RASSF1A*, Hs00153890_m1 for *DIRAS3,* and Hs99999905-m1 for *GAPDH* (TaqMan, Applied Biosystems, CA, USA) were pre-loaded on the Micro Fluidic Cards.

The *AKAP9* expression was examined in 78 pairs of thyroid specimens with the KAPA SYBR FAST Universal qPCR containing SYBR Green I Dye (KAPA Biosystems, Wilmington, MA, USA), and reactions were performed in the ECO Real-Time PCR (Illumina, San Diego, CA, USA). The reaction mixture comprised: KAPA SYBR FAST qPCR MasterMix, 1 µL of cDNA, and 5.0 µM of forward and reverse primer (Sigma-Aldrich, MO, USA).

Data obtained from different types of thyroid lesions were normalized, and the expression level of target genes from pathologically changed with normal (unchanged) tissues was compared using the comparative 2^−ΔΔCt^ (RQ) method [67] in DataAssist™ v3.01 Software—Relative Quantification Assay software (Applied Biosystems, CA, USA) or with EcoStudy software V5.0 (Illumina, CA, USA).

### 4.5. GEO Dataset Analysis

The raw data of genes expression in thyroid tissue came from experiments using the Affymetrix GeneChip Human Genome U133/Plus 2.0 Array and were obtained from the Gene Expression Omnibus database (GEO; Series: GSE60542, GSE82208, GSE27155, GSE53157, GSE33630). The data were loaded into R studio using the *affy* package (https://doi.org/10.1093/bioinformatics/btg405 (accessed on 18 May 2023)) of the Bioconductor Repository (3.16) (https://doi.org/10.1093/bioinformatics/btm254 (accessed on 30 April 2023)).

Normalization for all the datasets was performed using the *expresso* function, in which background correction was performed using the robust multi-array average expression measure, along with normalization using *quantiles* and *medianpolish* as the summarization method. Finally, *RASSF1A*, *DIRAS3*, and *AKAP9* expressions were analysed in 227 pathologically changed and 80 normal thyroid tissue samples (including 44 patient-matched PTC and normal thyroid tissue samples) characterised in Table 4 and Table 5.

### 4.6. Statistical Analysis

The statistical analysis was performed using Statistica 13.1 software (StatSoft, Cracow, Poland) (v.13.1). Gaussian data distributions were tested with the Shapiro–Wilk test. The distribution of all obtained results departs from the Gaussian distribution. Thus, non-parametrical statistical tests were used: Mann–Whitney U-test (UMW) or Wilcoxon matched-pairs signed rank test for comparison between two non-paired or paired groups, respectively; Kruskal-Wallis test (KW) for multiple comparisons; and the Spearman’s rank correlation (rs). The results of the relative expression analysis (RQ value) are presented as median with interquartile range (IQR). One sample Wilcoxon test was used for comparison of RQ values with arbitrary expression level equal 1 (where gene expression in pathologically changed thyroid tissue was equal to expression in control sample). Multivariate analysis was performed using data from the *BRAF* V600E mutation and *RASSF1A* hypermethylation assessment performed in this study, as well as the raw data regarding LOH/MSI in chromosomal instabilities in *RASSF1A* and *DIRAS3* gene from the previous study [68].

Receiver operating characteristic (ROC) curve analyses were performed to assess the sensitivity and specificity of the studied genes in estimating the diagnostic value in the classification of thyroid malignancy. A measure of the overall performance of a diagnostic test is the area under the ROC curve (AUC), and the AUC was resolved with a 95% confidence interval (CI). The selection of the optimal cut-off point for differentiating histopathological subtypes of thyroid cancer was determined using the Youden index (J). J is the maximum vertical distance between the ROC curve and the diagonal reference line, and is calculated as J = maximum (sensitivity + specificity − 1) [69].

Statistical analysis of categorical data (presence of LOH/MSI or *BRAF* V600E mutations) was performed using the Chi-square test (χ^2^) and Fisher exact test.

Statistical significance was determined at the level of *p* < 0.05, where the three significance levels were established: * *p* = 0.05–0.01; ** *p* = 0.01–0.001; *** *p* < 0.001.

## 5. Conclusions

The presented results give reason to consider the potential role of assessment of *RASSF1A*, *DIRAS3*, and *AKAP9* expression as molecular factors supporting the *BRAF* V600E point mutation analysis in the improvement of preoperative diagnosis and differentiation of various types of thyroid tissue lesions.

Since the *BRAF* V600E mutation was more frequently detected in malignant tissues than in benign lesions, searching for its presence in pathologically changed thyroid tissue may be particularly useful as a differential marker referring the patient for surgery. However, our and other published results indicate the need to standardize the type of method used for *BRAF* V600E detection, and to select a method as sensitive and reliable as possible. The decreased expression of tumour suppressors *RASSF1A* and *DIRAS3* genes—broadly observed in cancerous tissues compared to normal thyroid tissues—proved its involvement in thyroid carcinogenesis. The detection of decreased expression in both *RASSF1A* and *DIRAS3* holds particular significance as characteristic features of follicular-type changes (FTC and FA). This finding can serve as a marker for differentiating PTC from follicular-type thyroid tissue lesions. Our research also demonstrates for the first time that the alteration of *AKAP9* gene expression plays a role in the thyroid cancerogenesis; moreover, it indicates the development towards a follicular phenotype. Significantly increased expression of *AKAP9* in pathologically changed thyroid tissue and in more advanced cancer stages (pT2-4, Stage II-IV) may be potentially used as a thyroid tumorigenesis marker. Additionally, it may provide information on a more severe course of thyroid cancer.

## Figures and Tables

**Table 1:** Distribution of samples with *BRAF* V600E mutation among different clinical and pathological features of samples.

Clinical and Pathological Features of Samples		Number of Samples:		*p* ^#^
		*BRAF* V600E	*BRAF* WT	
A	Patient’s age (below vs over median)	16 vs 21	35 vs 31	0.412
B	Gender (women vs men)	33 vs 4	55 vs 11	0.564
C	Type of lesion (benign vs malignant)	11 vs 26	41 vs 28	** 0.004
D	Histopathological type (NG vs PTC)	7 vs 23	34 vs 20	*** 0.0006
E	Tumour diameter (below vs over median)	13 vs 12	21 vs 11	0.415
F	pT scale (pT1 vs pT2-4)	17 vs 9	15 vs 9	1.000
G	N (N0 vs N1)	18 vs 7	19 vs 3	0.297
H	Stage (AJCC I vs AJCC II-IV)	17 vs 8	16 vs 8	1.000

^#^ Fisher exact test; ** *p* = 0.01–0.001, *** *p* < 0.001.

**Table 2:** Clinical and pathological characteristics of the studied samples.

Clinical and Pathological Features	
**Number of sample pairs**	120
Median age (years) *	52.0 (36.2–60.7)
Gender	
Women	100
Men	20
**Histopathological type**	
NG	46
FA	14
FTC	11
PTC	49
Oxyphilic Metaplasia **	5

NG, nodular goitre; FA, follicular adenoma; PTC, papillary thyroid carcinoma; FTC, follicular thyroid carcinoma; * median (IQR); ** in NG group only.

**Table 3:** The histopathological verification of follicular-cell-derived thyroid tumours.

Clinical and Pathological Features	
**Number of sample pairs**	60
Median primary tumour diameter (mm) *	14.0 (7.7–25.0)
Below the median	31
Over the median	25
nd	4
**pTNM**	
T1	40
T2	5
T3	15
N0	44
N1	11
nd	5
**AJCC classification**	
AJCC I	41
AJCC II	3
AJCC III	12
AJCC IV	3
nd	1
**Tumour encapsulation**	
No	32
Yes	17
nd	11

pTNM, pathological tumour-node-metastasis classification; AJCC, American Joint Committee on Cancer; nd, no data in pathomorphological reports; * median (IQR).

**Table 4:** Clinical and pathological characteristics of the samples obtained from the GEO database.

Clinical and Pathological Features	
**Sample number**	307
Normal thyroid tissue	80
Pathologically changed tissue	227
Median age (years) */**	50.00 (36.00–62.25)
Below the median	53
Over the median	49
nd	125
**Gender**	
Women	92
Men	41
nd	172
**Histopathological type**	
Normal thyroid tissue	80
FA	35
FTC	44
PTC	148

FA, follicular adenoma; PTC, papillary thyroid carcinoma; FTC, follicular thyroid carcinoma; * median (IQR); ** data available in reports for 132 samples; nd, no data available.

**Table 5:** The histopathological characteristic of thyroid tumours in analysed GEO datasets.

Clinical and Pathological Features	
**Sample number**	192
Median primary tumour diameter (mm) *	1.60 (1.50–3.00)
Below the median	16
Over the median	15
Nd	161
**pTNM**	
T1	6
T2	2
T3	22
T4	2
nd	160
N0	14
N1	19
nd	159
**AJCC classification**	
AJCC I	19
AJCC II	-
AJCC III	10
AJCC IV	3
nd	160

pTNM, pathological tumour-node-metastasis classification; AJCC, American Joint Committee on Cancer; nd, no data available; * median (IQR).

## Data Availability

In our study, we analysed publicly available datasets obtained from the Gene Expression Omnibus database (GEO; Series: GSE60542, GSE82208, GSE27155, GSE53157, GSE33630). Direct links are provided below: https://www.ncbi.nlm.nih.gov/geo/query/acc.cgi?acc=GSE60542 (accessed on 18 May 2023) https://www.ncbi.nlm.nih.gov/geo/query/acc.cgi?acc=GSE82208 (accessed on 18 May 2023) https://www.ncbi.nlm.nih.gov/geo/query/acc.cgi?acc=GSE27155 (accessed on 18 May 2023) https://www.ncbi.nlm.nih.gov/geo/query/acc.cgi?acc=GSE53157 (accessed on 18 May 2023) https://www.ncbi.nlm.nih.gov/geo/query/acc.cgi?acc=GSE33630 (accessed on 18 May 2023). Data generated in this study, based on GEO datasets and raw data obtained from our experiments, will be made available on request.

## Supplementary Materials

The supporting information can be downloaded at: https://www.mdpi.com/article/10.3390/ijms25010562/s1.

## References

[1] Kebebew E., Weng J., Bauer J., Ranvier G., Clark O.H., Duh Q.Y., Shibru D., Bastian B., Griffin A. (2007). The prevalence and prognostic value of BRAF mutation in thyroid cancer. Ann. Surg..

[2] Lee S.T., Kim S.W., Ki C.S., Jang J.H., Shin J.H., Oh Y.L., Kim J.W., Chung J.H. (2012). Clinical implication of highly sensitive detection of the BRAF V600E mutation in fine-needle aspirations of thyroid nodules: A comparative analysis of three molecular assays in 4585 consecutive cases in a BRAF V600E mutation-prevalent area. J. Clin. Endocrinol. Metab..

[3] Li M., Dal Maso L., Vaccarella S. (2020). Global trends in thyroid cancer incidence and the impact of overdiagnosis. Lancet Diabetes Endocrinol..

[4] Sung H., Ferlay J., Siegel R.L., Laversanne M., Soerjomataram I., Jemal A., Bray F. (2021). Global Cancer Statistics 2020: GLOBOCAN Estimates of Incidence and Mortality Worldwide for 36 Cancers in 185 Countries. CA Cancer J. Clin..

[5] Pizzato M., Li M., Vignat J., Laversanne M., Singh D., La Vecchia C., Vaccarella S. (2022). The epidemiological landscape of thyroid cancer worldwide: GLOBOCAN estimates for incidence and mortality rates in 2020. Lancet Diabetes Endocrinol..

[6] Patel H.H., Goyal N., Goldenberg D. (2014). Imaging, genetic testing, and biomarker assessment of follicular cell-derived thyroid cancer. Ann. Med..

[7] Guevara N., Lassalle S., Benaim G., Sadoul J.L., Santini J., Hofman P. (2015). Role of frozen section analysis in nodular thyroid pathology. Eur. Ann. Otorhinolaryngol. Head Neck Dis..

[8] Sakorafas G.H., Peros G., Farley D.R. (2006). Thyroid nodules: Does the suspicion for malignancy really justify the increased thyroidectomy rates?. Surg. Oncol..

[9] Muzza M., Colombo C., Pogliaghi G., Karapanou O., Fugazzola L. (2020). Molecular markers for the classification of cytologically indeterminate thyroid nodules. J. Endocrinol. Investig..

[10] Nylén C., Mechera R., Maréchal-Ross I., Tsang V., Chou A., Gill A.J., Clifton-Bligh R.J., Robinson B.G., Sywak M.S., Sidhu S.B. (2020). Molecular Markers Guiding Thyroid Cancer Management. Cancers.

[11] Rajab M., Payne R.J., Forest V.I., Pusztaszeri M. (2022). Molecular Testing for Thyroid Nodules: The Experience at McGill University Teaching Hospitals in Canada. Cancers.

[12] Xing M. (2007). BRAF mutation in papillary thyroid cancer: Pathogenic role, molecular bases, and clinical implications. Endocr. Rev..

[13] Schubert L., Mariko M.L., Clerc J., Huillard O., Groussin L. (2023). MAPK Pathway Inhibitors in Thyroid Cancer: Preclinical and Clinical Data. Cancers.

[14] Frasca F., Nucera C., Pellegriti G., Gangemi P., Attard M., Stella M., Loda M., Vella V., Giordano C., Trimarchi F. (2008). BRAF(V600E) mutation and the biology of papillary thyroid cancer. Endocr. Relat. Cancer.

[15] Huang T., Zhuge J., Zhang W.W. (2013). Sensitive detection of BRAF V600E mutation by Amplification Refractory Mutation System (ARMS)-PCR. Biomark Res..

[16] Schulten H.J., Alotibi R., Al-Ahmadi A., Ata M., Karim S., Huwait E., Gari M., Al-Ghamdi K., Al-Mashat F., Al-Hamour O. (2015). Effect of BRAF mutational status on expression profiles in conventional papillary thyroid carcinomas. BMC Genom..

[17] Brzeziańska E., Pastuszak-Lewandoska D., Wojciechowska K., Migdalska-Sek M., Cyniak-Magierska A., Nawrot E., Lewiński A. (2007). Investigation of V600E BRAF mutation in papillary thyroid carcinoma in the Polish population. Neuro Endocrinol. Lett..

[18] Chakraborty A., Narkar A., Mukhopadhyaya R., Kane S., D’Cruz A., Rajan M.G. (2012). BRAF V600E mutation in papillary thyroid carcinoma: Significant association with node metastases and extra thyroidal invasion. Endocr. Pathol..

[19] Ciampi R., Zhu Z., Nikiforov Y.E. (2005). BRAF copy number gains in thyroid tumors detected by fluorescence in situ hybridization. Endocr. Pathol..

[20] Amin K.S., Banerjee P.P. (2012). The cellular functions of RASSF1A and its inactivation in prostate cancer. J. Carcinog..

[21] Li X., Liu S., Fang X., He C., Hu X. (2019). The mechanisms of DIRAS family members in role of tumor suppressor. J. Cell Physiol..

[22] Dubois F., Bergot E., Zalcman G., Levallet G. (2019). RASSF1A, puppeteer of cellular homeostasis, fights tumorigenesis, and metastasis-an updated review. Cell Death Dis..

[23] García-Gutiérrez L., McKenna S., Kolch W., Matallanas D. (2020). RASSF1A Tumour Suppressor: Target the Network for Effective Cancer Therapy. Cancers.

[24] Bildik G., Liang X., Sutton M.N., Bast R.C., Lu Z. (2022). DIRAS3: An Imprinted Tumor Suppressor Gene that Regulates RAS and PI3K-driven Cancer Growth, Motility, Autophagy, and Tumor Dormancy. Mol. Cancer Ther..

[25] Sutton M.N., Lu Z., Li Y.C., Zhou Y., Huang T., Reger A.S., Hurwitz A.M., Palzkill T., Logsdon C., Liang X. (2019). DIRAS3 (ARHI) Blocks RAS/MAPK Signaling by Binding Directly to RAS and Disrupting RAS Clusters. Cell Rep..

[26] Yu Y., Luo R., Lu Z., Feng W.W., Badgwell D., Issa J.P., Rosen D.G., Liu J., Bast R.C. (2006). Biochemistry and biology of ARHI (DIRAS3), an imprinted tumor suppressor gene whose expression is lost in ovarian and breast cancers. Methods Enzymol..

[27] Lu Z., Luo R.Z., Lu Y., Zhang X., Yu Q., Khare S., Kondo S., Kondo Y., Yu Y., Mills G.B. (2008). The tumor suppressor gene ARHI regulates autophagy and tumor dormancy in human ovarian cancer cells. J. Clin. Investig..

[28] Badgwell D.B., Lu Z., Le K., Gao F., Yang M., Suh G.K., Bao J.J., Das P., Andreeff M., Chen W. (2012). The tumor-suppressor gene ARHI (DIRAS3) suppresses ovarian cancer cell migration through inhibition of the Stat3 and FAK/Rho signaling pathways. Oncogene.

[29] Lu Z., Baquero M.T., Yang H., Yang M., Reger A.S., Kim C., Levine D.A., Clarke C.H., Liao W.S., Bast R.C. (2014). DIRAS3 regulates the autophagosome initiation complex in dormant ovarian cancer cells. Autophagy.

[30] Czarnecka K., Pastuszak-Lewandoska D., Migdalska-Sek M., Nawrot E., Brzezinski J., Dedecjus M., Pomorski L., Brzezianska E. (2011). Aberrant methylation as a main mechanism of TSGs silencing in PTC. Front. Biosci. Elite Ed..

[31] Weber F., Aldred M.A., Morrison C.D., Plass C., Frilling A., Broelsch C.E., Waite K.A., Eng C. (2005). Silencing of the maternally imprinted tumor suppressor ARHI contributes to follicular thyroid carcinogenesis. J. Clin. Endocrinol. Metab..

[32] Reggi E., Diviani D. (2017). The role of A-kinase anchoring proteins in cancer development. Cell Signal..

[33] Colledge M., Scott J.D. (1999). AKAPs: From structure to function. Trends Cell Biol..

[34] Jo Y.S., Kim M.S., Yoo N.J., Lee S.H. (2016). Frameshift Mutations of AKAP9 Gene in Gastric and Colorectal Cancers with High Microsatellite Instability. Pathol. Oncol. Res..

[35] Ciampi R., Knauf J.A., Kerler R., Gandhi M., Zhu Z., Nikiforova M.N., Rabes H.M., Fagin J.A., Nikiforov Y.E. (2005). Oncogenic AKAP9-BRAF fusion is a novel mechanism of MAPK pathway activation in thyroid cancer. J. Clin. Investig..

[36] Yang M.H., Hu Z.Y., Xu C., Xie L.Y., Wang X.Y., Chen S.Y., Li Z.G. (2015). MALAT1 promotes colorectal cancer cell proliferation/migration/invasion via PRKA kinase anchor protein 9. Biochim. Biophys. Acta.

[37] Hu Z.Y., Liu Y.P., Xie L.Y., Wang X.Y., Yang F., Chen S.Y., Li Z.G. (2016). AKAP-9 promotes colorectal cancer development by regulating Cdc42 interacting protein 4. Biochim. Biophys. Acta.

[38] Yan Q., Wu Y., Li D., Li Y. (2022). A-Kinase Anchoring Protein 9 Promotes Gastric Cancer Progression as a Downstream Effector of Cadherin 1. J. Oncol..

[39] Wu S., Shen D., Zhao L. (2022). AKAP9 Upregulation Predicts Unfavorable Prognosis in Pediatric Acute Myeloid Leukemia and Promotes Stemness Properties via the Wnt/β-Catenin Pathway. Cancer Manag. Res..

[40] Xing M., Alzahrani A.S., Carson K.A., Viola D., Elisei R., Bendlova B., Yip L., Mian C., Vianello F., Tuttle R.M. (2013). Association between BRAF V600E mutation and mortality in patients with papillary thyroid cancer. JAMA.

[41] Chen X., Zhou Q., Wang F., Zhang F., Du H., Zhang Q., Wu W., Gong X. (2018). Value of *BRAF* V600E in High-Risk Thyroid Nodules with Benign Cytology Results. AJNR Am. J. Neuroradiol..

[42] Kim W.Y., Kim H., Hwang T.S., Oh S.Y. (2017). Comparison between Real-Time PCR and Pyrosequencing for Detection of BRAF V600E Mutation in Thyroid Fine-Needle Aspirates. Appl. Immunohistochem. Mol. Morphol..

[43] Ellison G., Donald E., McWalter G., Knight L., Fletcher L., Sherwood J., Cantarini M., Orr M., Speake G. (2010). A comparison of ARMS and DNA sequencing for mutation analysis in clinical biopsy samples. J. Exp. Clin. Cancer Res..

[44] Nikiforova M.N., Kimura E.T., Gandhi M., Biddinger P.W., Knauf J.A., Basolo F., Zhu Z., Giannini R., Salvatore G., Fusco A. (2003). BRAF mutations in thyroid tumors are restricted to papillary carcinomas and anaplastic or poorly differentiated carcinomas arising from papillary carcinomas. J. Clin. Endocrinol. Metab..

[45] Koziołek M., Bińczak-Kuleta A., Stepaniuk M., Parczewski M., Andrysiak-Mamos E., Sieradzka A., Safranow K., Osowicz-Korolonek L., Kiedrowicz B., Kram A. (2015). Frequency assessment of BRAF mutation, KRas mutation, and RASSF1A methylation in nodular goitre based on fine-needle aspiration cytology specimens Ocena częstości występowania mutacji genów BRAF, KRas oraz. Endokrynol. Pol..

[46] Czarniecka A., Rusinek D., Stobiecka E., Krajewska J., Kowal M., Kropińska A., Zebracka J., Kowalska M., Włoch J., Maciejewski A. (2010). Occurrence of BRAF mutations in a Polish cohort of PTC patients-preliminary results. Endokrynol. Pol..

[47] Murugan A.K., Qasem E., Al-Hindi H., Shi Y., Alzahrani A.S. (2016). Classical V600E and other non-hotspot BRAF mutations in adult differentiated thyroid cancer. J. Transl. Med..

[48] Wang Z., Chen J.Q., Liu J.L., Qin X.G. (2016). Clinical impact of BRAF mutation on the diagnosis and prognosis of papillary thyroid carcinoma: A systematic review and meta-analysis. Eur. J. Clin. Investig..

[49] Nakamura N., Carney J.A., Jin L., Kajita S., Pallares J., Zhang H., Qian X., Sebo T.J., Erickson L.A., Lloyd R.V. (2005). RASSF1A and NORE1A methylation and BRAFV600E mutations in thyroid tumors. Lab. Investig..

[50] Pastuszak-Lewandoska D., Kordiak J., Migdalska-Sęk M., Czarnecka K.H., Antczak A., Górski P., Nawrot E., Kiszałkiewicz J.M., Domańska D., Brzeziańska-Lasota E. (2015). Quantitative analysis of mRNA expression levels and DNA methylation profiles of three neighboring genes: FUS1, NPRL2/G21 and RASSF1A in non-small cell lung cancer patients. Respir. Res..

[51] Schagdarsurengin U., Gimm O., Hoang-Vu C., Dralle H., Pfeifer G.P., Dammann R. (2002). Frequent epigenetic silencing of the CpG island promoter of RASSF1A in thyroid carcinoma. Cancer Res..

[52] Hoque M.O., Rosenbaum E., Westra W.H., Xing M., Ladenson P., Zeiger M.A., Sidransky D., Umbricht C.B. (2005). Quantitative assessment of promoter methylation profiles in thyroid neoplasms. J. Clin. Endocrinol. Metab..

[53] Xing M. (2007). Gene methylation in thyroid tumorigenesis. Endocrinology.

[54] Huang G., Chen J., Zhou J., Xiao S., Zeng W., Xia J., Zeng X. (2021). Epigenetic modification and BRAF gene mutation in thyroid carcinoma. Cancer Cell Int..

[55] Kunstman J.W., Korah R., Healy J.M., Prasad M., Carling T. (2013). Quantitative assessment of RASSF1A methylation as a putative molecular marker in papillary thyroid carcinoma. Surgery.

[56] Brown T.C., Juhlin C.C., Healy J.M., Prasad M.L., Korah R., Carling T. (2014). Frequent silencing of RASSF1A via promoter methylation in follicular thyroid hyperplasia: A potential early epigenetic susceptibility event in thyroid carcinogenesis. JAMA Surg..

[57] Xing M., Cohen Y., Mambo E., Tallini G., Udelsman R., Ladenson P.W., Sidransky D. (2004). Early occurrence of RASSF1A hypermethylation and its mutual exclusion with BRAF mutation in thyroid tumorigenesis. Cancer Res..

[58] Brait M., Loyo M., Rosenbaum E., Ostrow K.L., Markova A., Papagerakis S., Zahurak M., Goodman S.M., Zeiger M., Sidransky D. (2012). Correlation between BRAF mutation and promoter methylation of TIMP3, RARβ2 and RASSF1A in thyroid cancer. Epigenetics.

[59] Lázcoz P., Muñoz J., Nistal M., Pestaña A., Encío I., Castresana J.S. (2006). Frequent promoter hypermethylation of RASSF1A and CASP8 in neuroblastoma. BMC Cancer.

[60] Bryś M., Migdalska-Sęk M., Pastuszak-Lewandoska D., Forma E., Czarnecka K., Domańska D., Nawrot E., Wilkosz J., Różański W., Brzeziańska E. (2013). Diagnostic value of DNA alteration: Loss of heterozygosity or allelic imbalance-promising for molecular staging of prostate cancers. Med. Oncol..

[61] Zhu H., Qu Y. (2020). Expression levels of ARHI and Beclin1 in thyroid cancer and their relationship with clinical pathology and prognosis. Oncol. Lett..

[62] Dai J., Chen Q., Li G., Chen M., Sun H., Yan M. (2022). DIRAS3, GPR171 and RAC2 were identified as the key molecular patterns associated with brain metastasis of breast cancer. Front. Oncol..

[63] Truong T., Sauter W., McKay J.D., Hosgood H.D., Gallagher C., Amos C.I., Spitz M., Muscat J., Lazarus P., Illig T. (2010). International Lung Cancer Consortium: Coordinated association study of 10 potential lung cancer susceptibility variants. Carcinogenesis.

[64] Frank B., Wiestler M., Kropp S., Hemminki K., Spurdle A.B., Sutter C., Wappenschmidt B., Chen X., Beesley J., Hopper J.L. (2008). Association of a common AKAP9 variant with breast cancer risk: A collaborative analysis. J. Natl. Cancer Inst..

[65] Jarząb B., Dedecjus M., Słowińska-Klencka D., Lewiński A., Adamczewski Z., Anielski R., Bagłaj M., Bałdys-Waligórska A., Barczyński M., Bednarczuk T. (2018). Guidelines of Polish National Societies Diagnostics and Treatment of Thyroid Carcinoma. 2018 Update. Endokrynol. Pol..

[66] Edge S.B., Compton C.C. (2010). The American Joint Committee on Cancer: The 7th edition of the AJCC cancer staging manual and the future of TNM. Ann. Surg. Oncol..

[67] Schmittgen T.D., Livak K.J. (2008). Analyzing real-time PCR data by the comparative C(T) method. Nat Protoc..

[68] Migdalska-Sęk M., Czarnecka K.H., Kusiński M., Pastuszak-Lewandoska D., Nawrot E., Kuzdak K., Brzeziańska-Lasota E. (2019). Clinicopathological Significance of Overall Frequency of Allelic Loss (OFAL) in Lesions Derived from Thyroid Follicular Cell. Mol. Diagn. Ther..

[69] Akobeng A.K. (2007). Understanding Diagnostic Tests 3: Receiver Operating Characteristic Curves. Acta Paediatr..

