# Supplementary material for: Expression of RASSF1A, DIRAS3, and AKAP9 Genes in Thyroid Lesions: Implications for Differential Diagnosis and Prognosis of Thyroid Carcinomas
Source: Int J Mol Sci. 2024 Jan 1;25(1):562. doi: 10.3390/ijms25010562 (PMC10778957; doi:10.3390/ijms25010562)
Supplement: Supplementary file 1 [file ijms-25-00562-s001.zip › ijms-2759182-supplementary/ijms-2759182-supplementary.pdf]

Table S1. The comparison of the results of cytological diagnoses of FNAB with the results of histopathological diagnoses obtained after surgery.

Bethesda category*	Cytological FNAB verification	N of cases	Histopathological verification obtained after surgery	% of confirmed diagnosis in histopathology (n)
II	Benign	16	7 NG, 7 FA, 2 PTC	43.8% (7)
IV	Follicular neoplasm/ suspicious for follicular neoplasm	44	29 NG, 6 FA, 2 FTC, 3 PTC	25.0% (11)
V	Suspicious for malignancy	8	2 NG, 4 PTC, 2 FTC	75.0% (6)
VI	Malignant	52	8 NG, 1 FA, 40 PTC, 3 FTC	82.7% (43)

*Bethesda System for Reporting Thyroid Cytopathology; NG, nodular goitre; FA, follicular adenoma; PTC, papillary thyroid carcinoma; FTC, follicular thyroid carcinoma; n, number of samples

Table *S*2. List of nucleotide sequences of primers used in the study with the chromosomal location of the analyzed genes.

Gene (locus)	Primer sequence 5`- 3`	Product length	Description
BRAF **(7q34)**	Fo: CTCTTCATAATGCTTGCTCTGATAG<br>Ro: GCCTCAATTCTTACCATCCAC	200 bp	*BRAF* wild type
	Fiwt: GTGATTTTGGTCTAGCTACAGT<br>Rimut: CCCACTCCATCGAGATTTCT	97 bp (Fiwt +Ro)<br>144 bp (Fo + Rimut)	*BRAF* V600E
AKAP9 **(7q21.2)**	F: AGCAAGAACAGTTGATTTTGGA<br>R: TGAAGGTGAAACCTTAGTGTGA	181 bp	*AKAP9* wild type
DIRAS3 **(1p31.3)**	F: ACATCTTTGGTTTGGATAGATG<br>R: CAAAACTGCACATTTTGCAC	289 bp	D1S2137 – STS marker for LOH/MSI analysis
	F: GGGCATTGTTTAGGGGTG<br>R: TAGTGGGCTTTACGTCTGC	291 bp	D1S368 – STS marker for LOH/MSI analysis
RASSF1A **(3p21.3)**	F_{meth}: ATATTTTTTCGATTTGGAGTTTTTTC<br>R_{meth}: CTACACTATAACCTACCCATCCTCG	215 bp	Primers for MS-PCR for amplification of methylated promoter region
	F_{unmeth}: TTTTTTTGATTTGGAGTTTTTTTGT<br>R_{unmeth}: TACACTATAACCTACCCATCCTCAC	211 bp	Primers for MS-PCR for amplification of unmethylated promoter region
	F: CCAGGGTCTGTGTGAATGTG<br>R: CCCACAGGAGGCATTCAG	147 bp	D3S3615 – STS marker for LOH/MSI analysis

Bp, base pairs; Meth, methylated primer; Unmeth, unmethylated primer; F, forward primer; R, reverse primer; Fo, universal forward primer; Ro, universal reverse primer; Fiwt, wild-type BRAF primer; Rimut, mutated BRAF primer

Method S1. Direct automated fluorescent sequencing

11 randomly selected samples harbouring *BRAF* V600E mutation and 1 sample with wild type BRAF variant examined previously by ARMS-PCR were subsequently submitted to direct sequencing. The PCR product were excised and purified from an agarose gel using AxyPrep™ DNA Gel Extraction Kit (Axygen Scientific, USA). The sequencing PCR was performed using the BigDye™ Terminator v3.1 Cycle Sequencing Kit (Applied Biosystems, USA) and products were purified using magnetic beads kit D-Pure (Nimagen, Netherlands). After purification the genotyping was performed in capillary electrophoresis on 3130xl Genetic Analyzer (Applied Biosystems, Hitachi, USA) and assessed using Sequencing Analysis Software (Applied Biosystems, Hitachi, USA), according to the manufacturer's protocol.

Method S2. Bisulfite conversion and methylation-specific PCR (MS-PCR)

Total DNA was subjected to bisulfite conversion performed using the commercially available EpiMark Bisulfite Conversion Kit (New England BioLabs, MA, USA), according to the manufacturer's protocol. The quality and quantity of DNA were spectrophotometrically assessed at 260/280 nm with a BioPhotometer™ plus spectrophotometer (Eppendorf, Hamburg, Germany).

RASSF1A promoter hypermethylation was analysed using Methylation Specific PCR (MS-PCR) in 104 pairs of thyroid specimens. MS-PCR was performed using 1 ug of bisulfite-converted DNA, Hot Start AmpliTaq Gold 360 Polymerase (5U/µl; Sigma-Aldrich, MO, USA) and two pairs of primers (10.0 µM each; specific for methylated and unmethylated material and designed using the online MethPrimer tool (methPrimer v1.1 beta, Li Lab, Department of Urology, USCF) according to the criteria described in our previous publication [25]). The chromosomal location of the analysed genes and nucleotide sequences of primers used in the study are listed in Table S2. Amplification products were separated on 2% agarose gels, stained with ethidium bromide (0.5 mg/mL), and visualized under UV light on DigiDoc-It Imaging System (Ultra-Violet Products Ltd, USA). In the case of samples where both alleles were present, we have recognized probes as partially methylated, as the DNA was obtained from tissue homogenate.

Method S3. Evaluation of the LOH/MSI in chromosomal regions: 1p31.2 and 3p21.3

Multivariate analysis was performed using data from *BRAF* V600E mutation and *RASSF1A* hypermethylation assessment performed in this study, as well as the raw data regarding LOH/MSI in chromosomal instabilities in *RASSF1A* and *DIRAS3* gene from the previous study [44]. In short, the microsatellite instability analysis was done for chromosomal regions: 1p31.2 and 3p21.3, harbouring *RASSF1A* and *DIRAS3* genes. The chromosomal location of the analysed regions and nucleotide sequences of primers used in the study are listed in Table S2. PCR was performed using the Hot Start AmpliTaq 360® Polymerase (5U/µl) and 5.0 µM of primers labelled with a fluorescent dye. The amplification products were genotyped in capillary electrophoresis on 3130xl Genetic Analyzer (Applied Biosystems, Hitachi, MA, USA) and assessed using Sequencing Analysis Software (Applied Biosystems, Hitachi, MA, USA), according to the manufacturer's protocol.

Result S1. *BRAF* point mutations

The presence of *BRAF* V600E point mutation was initially analysed using ARMS-PCR method. Of 37 samples harboring V600E point mutation observed in ARMS-PCR study, 11 were submitted to Sanger sequencing in order to confirm the results (1 sample with excluded *BRAF* V600E mutation was used as control sample). The presence of *BRAF* missense mutation T1799A (V600E) was confirmed for 3 samples from 11 (Figure S1). Samples with mutations, proved by direct sequencing, showed as well weaker signals of mutant-type alleles. In the case of 2 samples in direct sequencing instead of V600E mutation, the *BRAF* Heterozygous missense mutation A1802G (K601E) was observed (Figure S1b).

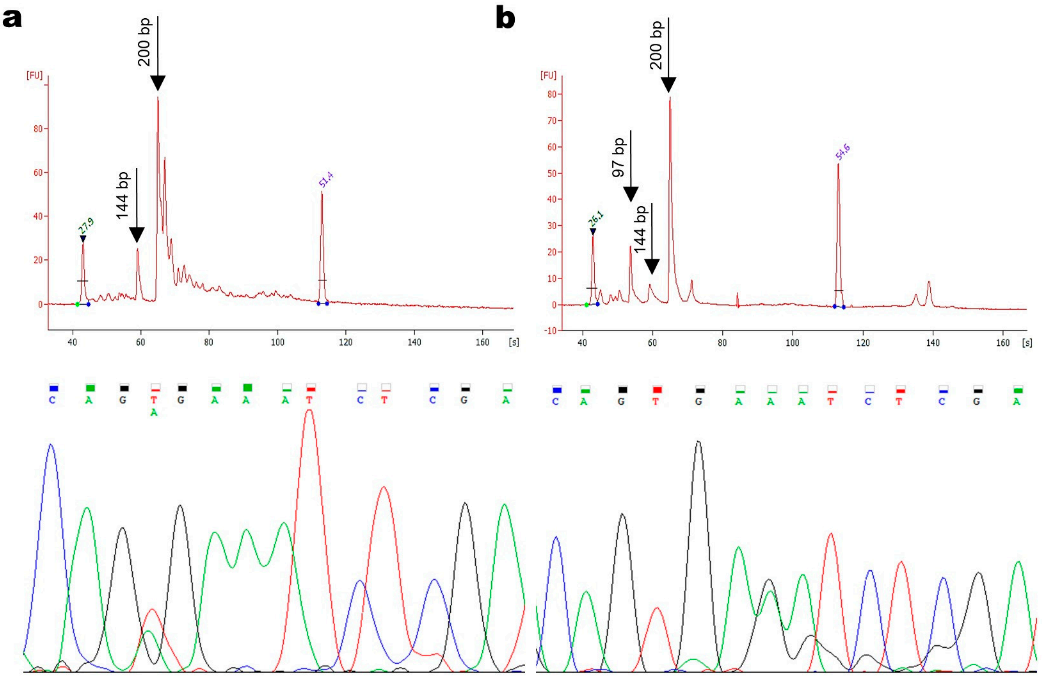

Figure S1. *BRAF* point mutations analysis by ARMS-PCR and direct sequencing. (a) Representative histogram (upper row) obtained from automated electrophoresis of sample with *BRAF* missense mutation (T1799A) detected in ARMS-PCR and confirmed in direct

sequencing (lower row); (b) Divergence of results obtained by ARMS-PCR (presence of *V600E* mutation; upper row) and direct sequencing (presence of *BRAF K601E* mutation, instead of *V600E*; lower row).

Insufficient number of samples did not allow for statistical analyzes of the obtained results, however, the distributions of indicated point mutations in different histopathological type of thyroid lesions are presented in Figure S2 and compared with the results of the ARMS-PCR analyzes.

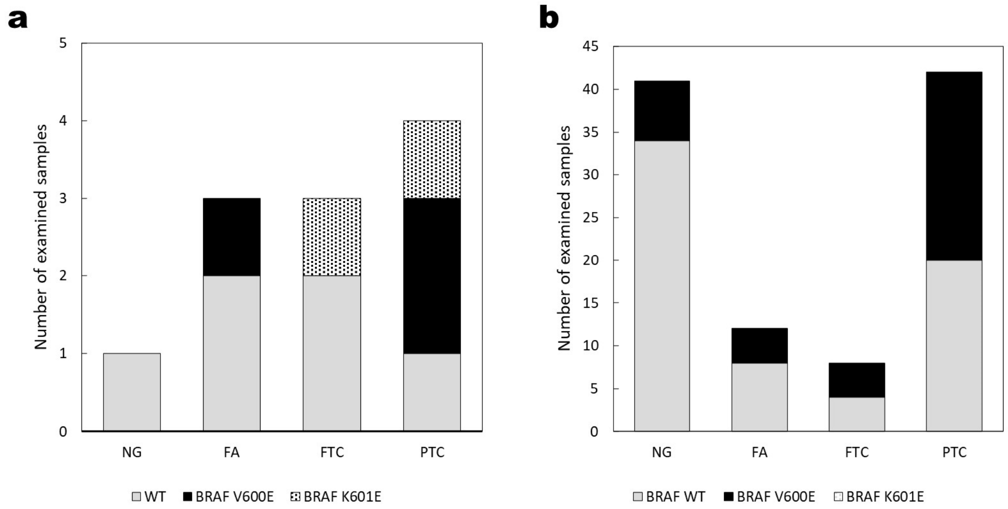

Figure S2. Comparison of distribution of different *BRAF* point mutations analysed by (a) direct sequencing and (b) ARMS-PCR.

Result S2. *RASSF1A* promoter hypermethylation

RASSF1A promoter hypermethylation (presence of methylated alleles) was observed in 35.6% samples of all the histopathological groups, with the most common being FA (53.8%) and FTC (45.4%). Of the remainder, hypermethylation was observed in PTC (31.8%) and non-cancerous lesions (NG, 30.6%). There was a tendency for more frequent hypermethylation observed in samples collected from patients with node invasion (N1 *vs* N0), although these differences were not statistically significant.

Result S3. Co-presence of mutations and genetic instabilities

Analyzing previously obtained data We assessed if LOH/MSI occurrence in 1p31.2 and 3p21.3 regions together with *BRAF* mutations and *RASSF1A* promoter hypermethylation leads to decreased expression of the genes of interest. The multivariable analysis showed that the presence of LOH/MSI in the *RASSF1A* region and *BRAF* V600E mutation both impact *RASSF1A* expression. In patients with malignant change, *BRAF* V600E mutation resulted in higher *RASSF1A* expression than those without BRAF mutation or with non-malignant change (p=0.048; Kruskal-Wallis rank sum test). The presence of LOH/MSI at more than one of the tested microsatellite sites was associated with increased *AKAP9* and decreased *DIRAS3* expression (p = 0.030 and p = 0.044, respectively, Kruskal-Wallis rank sum test).
